# Fermentation of Rice, Oat, and Wheat Flour by Pure Cultures of Common Starter Lactic Acid Bacteria: Growth Dynamics, Sensory Evaluation, and Functional Properties

**DOI:** 10.3390/foods13152414

**Published:** 2024-07-30

**Authors:** Konstantin V. Moiseenko, Olga A. Glazunova, Tatyana V. Fedorova

**Affiliations:** A. N. Bach Institute of Biochemistry, Research Center of Biotechnology, Russian Academy of Sciences, Leninsky Ave. 33/2, Moscow 119071, Russia; olga.a.glas@gmail.com (O.A.G.); fedorova_tv@mail.ru (T.V.F.)

**Keywords:** fermented cereals, cereal-based beverages, non-alcoholic fermentation, lactic acid bacteria, organoleptic evaluation, rheological properties, antioxidant capacity, angiotensin-converting enzyme inhibition

## Abstract

Recent consumer demand for non-dairy alternatives has forced many manufacturers to turn their attention to cereal-based non-alcoholic fermented products. In contrast to fermented dairy products, there is no defined and standardized starter culture for manufacturing cereal-based products. Since spontaneous fermentation is rarely suitable for large-scale commercial production, it is not surprising that manufacturers have started to adopt centuries-known dairy starters based on lactic acid bacteria (LABs) for the fermentation of cereals. However, little is known about the fermentation processes of cereals with these starters. In this study, we combined various analytical tools in order to understand how the most common starter cultures of LABs affect the most common types of cereals during fermentation. Specifically, 3% suspensions of rice, oat, and wheat flour were fermented by the pure cultures of 16 LAB strains belonging to five LAB species—*Lacticaseibacillus paracasei*, *Lactobacillus delbrueckii*, *Lactobacillus helveticus*, *Streptococcus thermophilus*, and *Lactococcus lactis*. The fermentation process was described in terms of culture growth and changes in the pH, reducing sugars, starch, free proteins, and free phenolic compounds. The organoleptic and rheological features of the obtained fermented products were characterized, and their functional properties, such as their antioxidant capacity and angiotensin-converting enzyme inhibitory activity, were determined.

## 1. Introduction

Non-alcoholic fermentation is an ancient food preservation process involving the biochemical transformation of food components under the action of enzymes without the formation of ethanol [1]. Enzymes are usually produced during the growth of particular microorganisms, although cell-free fermentation is possible in rare cases [2,3]. Currently, the number of different fermented foodstuffs known throughout the world is enormous. Without exaggeration, every extinct or existing culture have at least a dozen fermented products based on meat, fish, animal milk, fruits, vegetables, legumes, or cereals in its diet [4,5,6]. Today, many local, indigenous, and traditional non-alcoholic fermented foods have been commercialized and have truly become global culinary treasures [4,5]. Several examples of such foods are yogurt, soy sauce, vinegar, sauerkraut, kombucha, miso, kimchi, and sourdough bread [7,8].

Non-alcoholic fermentation is not only a relatively cheap and energy-effective way to ensure the shelf-life and microbiological safety of a product, it also drastically changes the product’s organoleptic characteristics, nutrient content, and health-related properties. During non-alcoholic fermentation, fermenting microorganisms can enrich the resulting product with vitamins, essential amino acids, polysaccharides, and proteins [3]. Also, fermentation can enhance the bioavailability of micronutrients, improve the digestibility of food fibers, and promote the degradation of anti-nutritional factors; the latter two points are especially important for cereal and plant-based foods, contributing to about 50% of the total dietary fiber intake in Western countries [9] and containing antinutrients such as phytates, tannins, saponins, and lectins [10]. Moreover, many microorganisms used for food fermentation have pronounced probiotic properties and, upon regular consumption, can provide health benefits such as the stimulation of immunity, restoration of the healthy gut microbiota, and even the alleviation of anxiety and depressive-like behaviors [11,12].

Lactic acid bacteria (LABs) are one of the most widespread groups of microorganisms used for food fermentation [13]. Although LABs are best known as major players in animal milk fermentation, they are an integral part of the bacterial consortia in spontaneously fermented fruits, vegetables, and cereals [14,15]. Currently, many pure cultures of LABs are sold commercially for the preparation of yogurt, cheese, butter, sour cream, etc. [16,17,18]. Also, LABs are archetypal probiotics. Since the discovery of the first probiotic microorganism (*Lactobacillus delbrueckii* subsp. bulgaricus) in 1905 by Stamen Grigorov [19,20], a huge amount of information has been collected and an incredible number of LAB strains with probiotic properties have been reported [21,22,23]. To date, more than 500 probiotic-related patents have been granted approval in the USA and Europe [24].

Besides exerting direct health-promoting effects as probiotics, LABs also produce a number of compounds that can provide health benefits to consumers. One of the most prominent types of such compounds is bioactive peptides [25,26]. By using proteins in the fermented foodstuffs, the proteolytic system of LABs generates a variety of peptides, some of which have valuable biological effects on human health [27]. The most well-known effects of these so-called “bioactive peptides” are antihypertensive, antidiabetic, antioxidant, immunomodulatory, and antimicrobial [28,29,30]. In general, the proteolytic activity of LABs is species- and strain-dependent, since each LAB species contains different types of proteinases, the number and identity of which may vary among strains [31]. Currently, fermentation is the most cost-effective way of producing bioactive peptides. Moreover, fermented food is a natural delivery system for such peptides [30,32,33].

Although fermented dairy products have firmly taken their place in the global market, recently consumer demand for their non-dairy alternatives has been constantly growing [15,34]. In particular, cereal-based non-alcoholic beverages have become increasingly accepted substitutes for their milk-based counterparts. As plant matrices, fermented cereal products are ideal for people on a low-fat diet and indispensable for people with lactose intolerance and casein allergies [35,36,37,38]. The fermentation of cereals can not only enrich them with amino acids and proteins but can also make the final product an excellent vehicle for delivering probiotic microorganisms and functional compounds [15,35,38].

Many previous studies on the fermentation of cereals have used spontaneous fermentation [39,40,41]. Although this method of fermentation mimics the original preparation of the products in rural areas, it is not strictly repeatable and, consequently, cannot directly benefit the food industry. The use of standardized starter cultures would allow quality control of the fermentation process, would facilitate the scale-up of the process, and would provide easy optimization of the variation in fermented raw material [40,42,43,44]. Moreover, it is possible that the use of different strains of LABs will lead to significantly different organoleptic and health-promoting properties of the resulting fermentation products.

Therefore, the objective of this article is to describe the fermentation process of three types of cereals—rice, oat, and wheat—by sixteen strains of LABs belonging to five LAB species, namely *Lacticaseibacillus paracasei*, *Lb. delbrueckii*, *Lactobacillus helveticus*, *Streptococcus thermophilus*, and *Lactococcus lactis*. All used LAB species are well-known and widely used starter cultures for fermented dairy products, while rice, oat, and wheat are promising ingredients for the production of cereal-based non-alcoholic beverages. For the description of the fermentation process, parameters such as the viable cell count, pH, content of reducing sugars, content of starch, content of free proteins, and content of free phenolic compounds were measured. The obtained fermented products were characterized in terms of their organoleptic and rheological properties as well as their antioxidant capacity and angiotensin-converting enzyme inhibitory activity.

## 2. Materials and Methods

### 2.1. Cereal Flour Used for Fermentation

Commercial rice (*Oryza sativa* L.), oat (*Avena sativa* L.), and wheat (*Triticum aestivum* L.) flours were purchased from a local supplier (Vkusvill, Moscow, Russia). The main composition of each flour is presented in Table 1.

### 2.2. Strains and Cultivation Conditions

The strains of LABs were obtained from the Collection of the All-Russian Research Institute of the Dairy Industry (VNIMI, Moscow, Russia), where they were stored as lyophilized cells at −80 °C. The main characteristics of each strain are presented in Table 2.

To obtain the starting inoculum, the lyophilized culture was added into the appropriate culture medium. The strains of *Lb. paracasei*, *Lb. delbrueckii*, and *Lb. helveticus* were cultivated overnight in MRS broth medium (HiMedia Laboratories, Mumbai, India) at 37 °C; the strains of *Str. thermophilus* were cultivated overnight in M17 broth medium (HiMedia Laboratories, Mumbai, India) at 37 °C; and the strains of *Lc. lactis* were cultivated overnight in M17 broth medium (HiMedia Laboratories, Mumbai, India) at 30 °C.

The starting flour suspensions were prepared as follows: 30 g of each flour (rice, oat, or wheat) was suspended in 970 mL of distilled water; then, the suspension was stirred for 60 min at 90 °C, autoclaved at 121 °C for 30 min, and re-autoclaved after 3 days under the same conditions.

The obtained starting suspensions of flour were inoculated with 1% (*v/v*) of the appropriate LAB starting inoculum. Fermentation with *Lb. paracasei*, *Lb. delbrueckii*, or *Lb. helveticus* was carried out at 37 °C, and fermentation with *Lc. lactis* was carried out at 30 °C. During fermentation, the samples were collected after 6, 12, 24, and 48 h. The starting suspensions of flour without fermentation were used as a control.

The pH value of each sample was measured using a pH meter (Mettler Toledo, Griefensee, Switzerland) at room temperature (20 ± 2 °C).

To determine the viable cell count of the studied LABs, the LABs were cultivated on the appropriate solid medium in anaerobic conditions at 37 °C in the Anaero Bag System 24 (HiMedia Laboratories, Mumbai, India), as described by Glazunova et al. [45]. The strains of *Lb. paracasei*, *Lb. delbrueckii*, and *Lb. helveticus* were cultivated on MRS agar medium (HiMedia Laboratories, Mumbai, India), and the strains of *Str. thermophilus* and *Lc. lactis* were cultivated on M17 agar medium (HiMedia Laboratories, Mumbai, India).

### 2.3. Biochemical Assays

After fermentation, 50 mL of each sample was centrifuged at 4000× *g* for 20 min at 4 °C (Eppendorf centrifuge 5702 R, Hamburg, Germany), and the supernatant was filtered through a 0.45 µm syringe filter with hydrophilic membrane (Merk Millipore, Darmstadt, Germany).

In the obtained supernatant, the content of reducing sugars was measured using the modified Somogyi–Nelson assay [46]—0.2 mL of the sample was mixed with 0.2 mL of Somogyi reagent and incubated at 100 °C for 1 h; sequentially, 0.2 mL of Nelson reagent, 0.4 mL of acetone, and 1 mL of distilled water were added. Adsorption was measured at 610 nm. The calibration curve was constructed with glucose.

The content of free phenolic compounds was measured with the Folin–Ciocalteu method [47,48]—100 µL of the sample was mixed with 500 µL of Folin–Ciocalteu (10%) and incubated for 8 min at room temperature; sequentially, 400 µL of sodium carbonate (7.5%) was added, and the mixture was incubated at room temperature for 60 min. Absorbance was measured at 765 nm. The calibration curve was constructed with gallic acid.

The degree of proteolysis was measured using the TNBS (2,4,6-trinitrobenzenesulfonic acid) method, as described by Begunova et al. [49]—0.25 mL of the sample and 2 mL of TNBS (Sigma-Aldrich, St. Louis, MO, USA) reagent (0.1% *w/v* in water) were mixed with 2 mL of sodium phosphate buffer (0.2 M, pH 8.2) and incubated at 50 °C for 60 min; the reaction was stopped with the addition of 4 mL of HCl (0.1 M), and absorbance was measured at 340 nm. The calibration curve was constructed with L-leucine (L-Leu), and the proteolytic activity was reported as L-Leu molar equivalents per g of protein: µmol (LE)·g^−1^ (Protein).

The antioxidant capacity was determined using the Trolox equivalent antioxidant capacity (TEAC) assay, as described by Moiseenko et al. [50]—20 µL of the sample and 180 µL of 2,2′-azinobis-(3-ethylbenzothiazoline-6-sulfonate radical cation (ABTS^•+^)) were mixed and incubated at room temperature for 40 min. The ABTS^•+^ was prepared by incubating a solution containing 7 mM ABTS (Sigma-Aldrich, St. Louis, MO, USA) and 2.45 mM potassium peroxodisulfate (Sigma-Aldrich, St. Louis, MO, USA) in the dark at room temperature for 12–18 h. Absorbance was measured at 734 nm. The antioxidant capacity was expressed as the amount of Trolox (Sigma-Aldrich, St. Louis, MO, USA) molar equivalents per g of protein: µmol (TE)·g^−1^ (Protein).

The angiotensin-converting enzyme (ACE) inhibitory activity was measured using ACE (Sigma-Aldrich, St. Louis, MO, USA) and an o-Aminobenzoyl-Phe-Arg-Lys(dinitrophenyl)-Pro (Sigma-Aldrich, St. Louis, MO, USA) substrate, as described in Kruchinin et al. [51].

The starch content of the flour suspensions before and after fermentation was determined using a Total Starch Assay Kit (K-TSTA-100A, Megazyme International Ireland Ltd., Bray, Ireland) according to either of the following protocols: (a) “The Rapid Total Starch (RTS) Method”, or (b) the “Determination of total starch content of samples containing resistant starch” method, provided by the manufacturer.

### 2.4. Organoleptic Assessment

All products were stored for 1 day at 5 °C before the sensory evaluation. The sensory evaluation was carried out using 16 panelists familiar with the product. The samples were evaluated for consistency, flavor, taste, color, and overall acceptability, on a five-point hedonic scale: one point—dislike extremely, two points—dislike slightly, three points—neither like nor dislike; four points—like slightly, and five points—like extremely. All panelists received written information about the test and all of the participants provided their informed consent to participate in the study. The study was conducted according to the guidelines of the Declaration of Helsinki, and approved by the Ethics Committee of the Federal Research Center Fundamentals of Biotechnology of the Russian Academy of Sciences (Approval No. 13 from 24 September 2023).

### 2.5. Rheological Measurements

The dynamic viscosity of the samples was measured using an LVDV-II+Pro rotation viscometer (Brookfield Engineering Laboratories Inc., Middleboro, MA, USA) equipped with a spindle (#61) and a special beaker. The measurements were carried out at room temperature (20 ± 2 °C); the values were recorded while increasing the shear rate from 12.9 to 64.6 s^−1^. The shear rate is reported in s^−1^ after multiplying the rpm by a conversion factor of 1.29 s^−1^, as specified by the manufacturer. All measurements were carried out in triplicate.

### 2.6. Statistical Analyses

All statistical comparisons were firstly performed using a one-way ANOVA omnibus *F*-test. When a significant (*p* < 0.05) value of the *F*-statistic was found, differences between means were evaluated using Tukey’s HSD (honestly significant difference) multiple-comparison test (*p* < 0.05).

## 3. Results and Discussion

### 3.1. Cell Growth, pH, and Concentration of Reducing Sugars

For fermentation, a 3% mixture of rice, oat, or wheat flour in water was inoculated with one of the 16 studied LAB strains. The started inoculum was in the range of 10^5^–10^7^ CFU·mL^−1^. Before proceeding with a detailed description of the dynamics of the fermentation process, it is necessary to highlight one important feature of the fermentation of cereal flours which was noted in our work and which should be taken into account when comparing our results with others. Flour contains a significant amount of endogenous microflora, to such an extent that it is even used as a starter in a number of traditional fermentations, such as in the fermentation of mahewu (a cornflour-based beverage whose fermentation is initiated by wheat flour) [39,52,53]. Not all bacteria associated with flour can be killed by simple one-step autoclaving, especially spore-forming bacteria. Therefore, a more complex substrate sterilization process—tyndallization-like—is required [54,55,56]. Ignoring this feature, together with neglecting to conduct a periodic microscopic assessment of culture purity, can lead to erroneous results regarding the number of viable LABs in fermented cereal flour. Some researchers even report 10^11^ CFU·mL^−1^ [57], a number that is almost impossible to obtain without enormous optimization efforts using nutrient-rich media [58].

**Figure 1:**
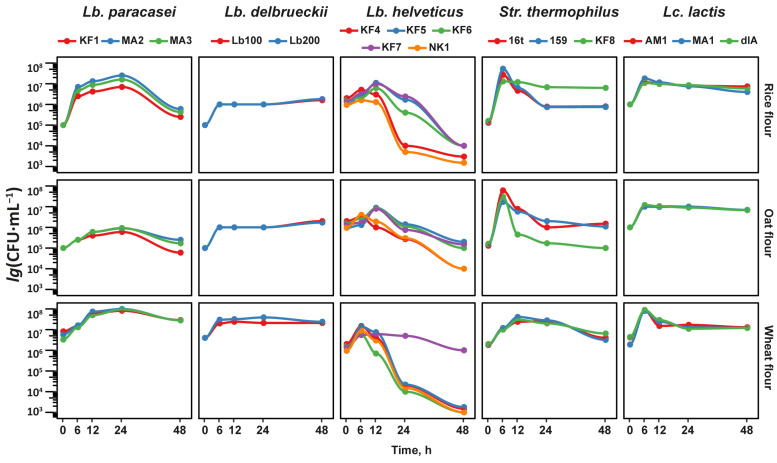
Growth curves of the studied LAB strains during the fermentation of cereals.

**Figure 2:**
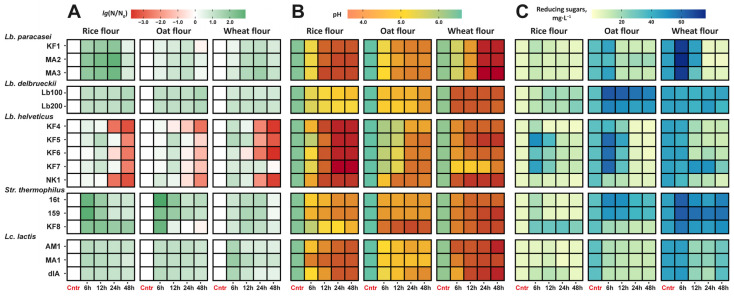
Changes in the relative viable cell count (**A**), pH (**B**), and concentration of reducing sugars (**C**) during the fermentation of cereals with the studied LAB strains.

The growth curves obtained during the fermentation of different cereals by the studied LABs are presented in Figure 1 (and the relative changes in the number of viable cells are presented in Figure 2A to facilitate comparison with changes in the pH values and amount of reducing sugars). In general, the growth patterns of the studied LABs were species-specific, and strain-specific deviations from the main pattern were rare. For all LABs, an increase of one to two orders of magnitude in the viable cell count was observed during the first 6–12 h of fermentation, followed by an approximately one-order-of-magnitude decrease for the strains of *Lb. paracasei*, a stationary phase for the strains of *Lb. delbrueckii* and *Lc. lactis*, and sharp decreases of three to four orders of magnitude for the strains of *Lb. helveticus* and *Str. thermophilus*. Although in some cases the LABs were able to reach the maximum attainable cell count of 10^8^ CFU·mL^−1^, in most cases, the cell count did not exceed 10^7^ CFU·mL^−1^. Significant deviation from the growth of other strains of the same species were observed only for *Lb. helveticus* KF7 in the wheat flour and *Str. thermophilus* KF8 in the rice flour. Instead of sharply decreasing, the viable cell count of these strains remained at the same level after 6 h of fermentation. Also, *Str. thermophilus* KF8 demonstrated a one-order-of-magnitude-sharper decrease in the viable cell count after 6 h of its fermentation of oat flour compared to the other strains of *Str. thermophilus*.

Discussing the growth curves of the studied LABs, it should be noted that LABs are fastidious organisms that lack many metabolic pathways [59]; all LABs require a rich environment that contains compounds such as amino acids, peptides, vitamins, and nucleic acids for their growth [7,60,61]. As a substrate, cereals are characterized by low levels of readily available sugars and proteins [34]. Consequently, LABs cannot actively increase their abundance early in cereal fermentation due to the need to adapt to nutrient-poor substrates. In contrast, during milk fermentation, the same strains of LABs were able to reach 10^8^–10^9^ CFU·mL^−1^ during the first 12 h of fermentation [45].

During growth, all studied LABs were able to gradually acidify their fermentation medium, and the minimal attainable pH level was reached during the first 12–24 h of fermentation, regardless of the cereal flour used for the medium preparation (Figure 2B). Based on the acidification pattern, all studied cereal-based substrates can be ranked from the most to the least easily acidified as follows: wheat flour ≥ rice flour > oat flour. Since this trend was observed for almost all studied LABs, it probably reflects the different buffer capacities of the used flours. Among all studied LABs, the highest acidification ability was demonstrated by *Lb. paracasei* and *Lb. helveticus*; these strains were able to acidify the rice and wheat flour media to 3.9 ± 0.2 pH and the oat flour medium to 4.5 ± 0.2 pH. The lowest acidification ability was demonstrated by the strains of *Lb. delbrueckii*. These strains were able to acidify rice and oat flour media only to 4.8 ± 0.4 pH; however, for wheat flour, the lowest attainable pH was comparable to that found for the other species of LABs and comprised 4.2 pH. Interestingly, the LAB strains that showed significant deviations from the overall species-specific pH trend with certain substrates were the same strains that showed deviant growth patterns in those substrates (Figure 1). The strains *Lb. helveticus* KF7 in wheat flour and *Str. thermophilus* KF8 in rice flour acidified their substrates significantly less compared to other strains of the same species, and *Str. thermophilus* KF8 was the most acidifying strain for the oat flour.

Acidification of the fermented medium is an important factor influencing the growth of LABs. Since all studied LABs are either obligate homo-fermenters or facultative hetero-fermenters, the main end product of their sugar metabolism is lactic acid [7,62]. During fermentation, the accumulation of lactic acid eventually leads to depletion of the buffer capacity and acidification of the medium. At pH values lower than four, lactic acid mainly takes on an undissociated form that is highly soluble in phospholipid cell membranes and can easily enter cells through passive diffusion [63]. The dissociation of the lactic acid inside cells leads to acidification of the cytosol, osmotic stress, metabolic permutations, and the dissipation of membrane potential [64,65]. In general, the ability to withstand acidic stress is supposed to be a strain-specific feature of LABs [66]; however, in our study, the LABs demonstrated rare strain-specific deviations in their growth patterns (Figure 1). It has previously been suggested that some probiotic-related traits of LABs may be widespread among most strains of the genus rather than being strain-specific [67]. This may also be true for stress-related traits, especially complex traits that involve many interconnected genes, and the ability to tolerate acidic stress is such a multigenic trait [65,68].

To assess the equilibrium between the hydrolysis and consumption of carbohydrates by the studied LABs, the amount of reducing sugars in the fermentation medium was measured (Figure 2C). It should be noted that at the beginning of fermentation, the amount of reducing sugars was significantly (*p* < 0.05) different in all fermented flours and comprised 5 ± 2, 33 ± 4, and 45 ± 5 mg·L^−1^ in the rice, oat, and wheat flours, respectively. At the 6 h point of fermentation, the following changes were detected: almost all strains of *Lb. paracasei* and *Str. thermophilus* had significantly (*p* < 0.05) increased the amount of reducing sugars in all fermentation media; almost all strains of *Lb. helveticus* had significantly (*p* < 0.05) increased the amount of reducing sugars in the rice and oat flour; and all strains of *Lc. lactis* had significantly (*p* < 0.05) decreased the amount of reducing sugars in the oat flour. During further fermentation, the amount of reducing sugars correlated very well with the overall growth patterns (Figure 1 and Figure 2A). The LABs that decreased the number of viable cells also decreased the amount of reducing sugars, and the LABs that did not significantly change the number of viable cells did not significantly change the amount of reducing sugars.

Previous transcriptomic and proteomic studies have highlighted that under certain stress conditions (i.e., acidic, thermal, and osmotic stresses), many LABs increase their levels of glycolytic enzymes [65]. Also, acidic stress leads to more rigid cell membranes, which hinders the secretion of proteins [64,65]. Therefore, it can be assumed that for those LABs that are less resistant to acidic stress, the decrease in reducing sugars can be explained by an increase in the production of glycolytic enzymes. At the same time, the production of glycoside hydrolases stops under acidic stress, possibly due to difficulty in their secretion.

### 3.2. Content of Starch, Free Proteins, and Free Phenolic Compounds

**Figure 3:**
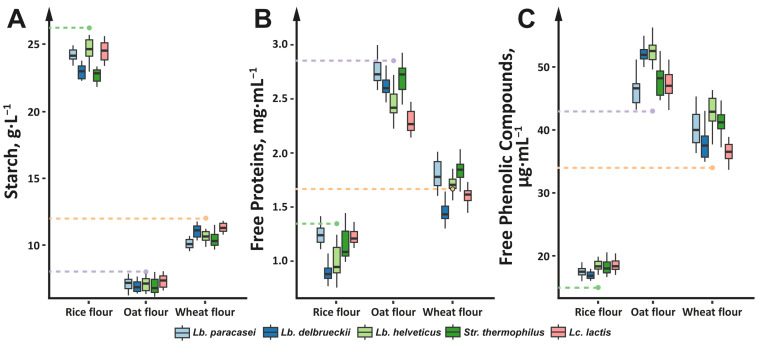
Changes in starch (**A**), free protein (**B**), and free phenolic compound (**C**) contents induced by the fermentation of cereal flours with the studied LAB strains. All measured values (for all studied LABs at all time points) are pooled together. Values measured for the unfermented flour are represented by dashed lines with a dot at the end.

In contrast to the pH and reducing sugars, the contents of starch, free proteins, and free phenolic compounds did not correlate with the growth patterns of the LABs. For all fermentations, their values significantly (*p* < 0.05) changed in the first 6 h, after which no changes were detected. Moreover, the magnitude of changes in each substrate was independent of the LAB strain used for fermentation. Therefore, for each substrate, all of the measured values (for all studied LABs at all time-points) were pooled together for graphical presentation (Figure 3) and further discussion. In general, fermentation of the cereal flours by the studied LABs resulted in a significant (*p* < 0.05) increase in the free phenol content and a significant (*p* < 0.05) decrease in the starch and free protein contents; the only exception was the fermentation of wheat flour, where the free protein content did not change during fermentation.

As previously mentioned, LABs are fastidious organisms, and in order to obtain amino acids that they are unable to synthesize, LABs must actively digest proteins from their immediate environment [69,70]. The overall decrease in free protein content is likely the result of a higher rate of water-soluble protein consumption by the studied LABs relative to the rate of their release from flour particles. Typically, rice flour contains from 8 to 9% of proteins, of which albumins (i.e. water-soluble proteins according to the Osborn’s classification [71]) comprise 5–11%; oat flour contains approximately 16% of proteins, of which albumins comprise 10–20%; and wheat flour contains from 9 to 14% of proteins, of which albumins comprise 9–15% [72,73,74,75]. However, not all of these proteins can be immediately released into the aqueous environment because they are trapped within the flour particles [76,77]. For the wheat flour, the lack of change in the free protein content during the fermentation was more likely related to a greater degree of loosening in the structure of flour particles due to starch hydrolysis, which leads to the release of albumins into the environment at the same rate as its consumption by the LABs.

Similar to the free protein content, the overall increase in free phenolic content is also the result of a dynamic equilibrium established during fermentation. The major phenolic compounds of cereals are phenolic acids, flavonoids, and tannins [78]. Phenolic acids and flavonoids are relatively small molecules that contains from one to three aromatic rings, respectively. In contrast, tannins are high-molecular-weight polymeric phenolic compounds. It was previously reported that fermentation can have multiple opposing effects on the content of free phenolic compounds [78,79,80,81]. Processes such as the depolymerization of tannins, oxidation of hydrophobic phenolic compounds, breaking of bonds between phenolic compounds and various macromolecules, and loosening of the starch network promote the release of bound phenolic compounds into a free form. On the other hand, processes such as degradation and metabolization by LABs or binding to other components released during fermentation lead to a decrease in the content of free phenolic compounds. The overall increase in free phenolic compounds observed in our study is promising for future use of the studied LAB strains in the food industry, as free phenolic compounds may have multiple health-promoting properties, including antioxidant, anti-inflammatory, and hypocholesterolemic effects [82,83,84].

The changes in the content of starch during the fermentation of cereal flour by the studied LAB strains demand a special discussion. The data on the decrease in starch content reported in Figure 3A are related to the total starch, which includes both resistant and easily hydrolysable starch, since the protocol used for the starch quantification contained a NaOH treatment step (protocol (b)) “Determination of total starch content of samples containing resistant starch (RTS-NaOH Procedure)” with the used K-TSTA-100A “Total starch (α-amylase/amyloglucosidase) assay protocol” Megazyme kit), which increases the solubilization of retrograded amylose and promotes the hydrolysis of the resistant starch [85]. Interestingly, when the starch quantitation procedure was performed without the NaOH treatment step (protocol (a)) “The Rapid Total Starch (RTS) Method” with the K-TSTA-100A “Total starch (α-amylase/amyloglucosidase) assay protocol” Megazyme kit), a false increase in starch content was observed. A similar situation was previously described and meticulously studied by Zhang et al., who demonstrated that the amylose content first decreased then increased during the fermentation of proso millet flour [86]. In short, during fermentation, LABs can hydrolyze amylopectin from the crystalline region of starch granules into short-branch fragments, thereby increasing the content of easily hydrolysable starch. In general, the content of easily hydrolysable starch was increased from 12 ± 1, 5 ± 0.6, and 7± 0.5 g·L^−1^ in the control rice, oat, and wheat flours to 14 ± 1, 6 ± 0.3, and 11 ± 1 g·L^−1^, respectively. The wheat flour showing the largest increase in the content of easily hydrolysable starch compared to the other types of tested flour indirectly confirms our assumption of there being a greater degree of loosening of the structure of wheat flour particles during the fermentation process.

### 3.3. Organoleptic Properties

In terms of general appearance, color, flavor, and texture, all fermented cereal flours had a moderate degree of acceptance. In particular, all flour samples fermented by the strains of *Lb. paracasei* had an astringent taste with a slight bitterness, and all flour samples fermented by the strains of *Lb. helveticus* had a pronounced sour taste. The rice and wheat flours fermented by the strains of *Lc. lactis* had the greatest sweetness among others. Also, cereal flours fermented by the strains of *Str. thermophilus* possessed a characteristic refreshing fruity/floral aroma, and all flours fermented by the strains of *Lb. delbrueckii* and *Lb. helveticus* were noted to have an aroma typical for the crust of black Russian bread.

All fermented cereal flours had a much simpler taste and plain flavor compared to milk fermented by the same LAB strains [45]. This can be explained by the fewer compounds that can be metabolized by LABs into flavor-forming molecules. During milk fermentation, LABs typically produce such flavor-forming molecules as organic acids, aldehydes, ketones, and acetoin through metabolizing lactose, free amino acids, and citric acid [87,88], all of which are rarely found in cereals in quantities comparable to those found in milk [34,89]. In general, the plain flavor, without any particular notes and with moderate tartness, of typical cereal-based products [53] allows manufacturers to supplement them with natural or synthetic flavorings, since the possibility of adverse interactions occurring between the added and already-existing flavors is low.

### 3.4. Rheological Properties

**Figure 4:**
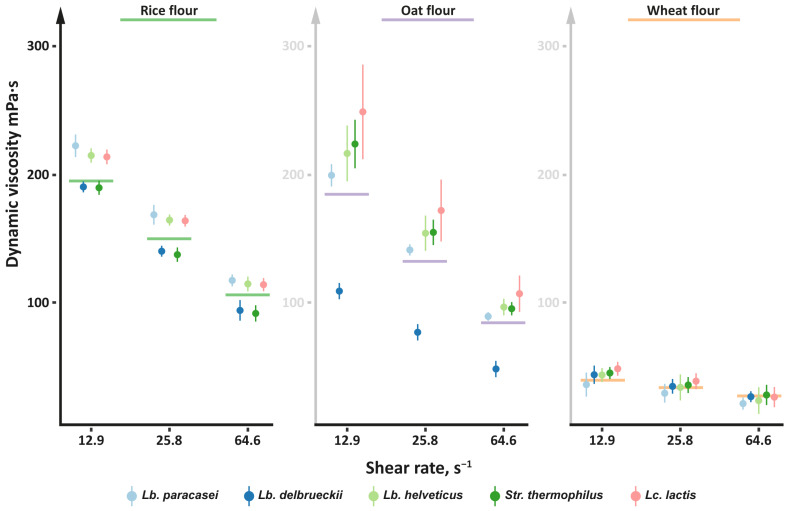
Changes in the dynamic viscosity of cereal flours after 24 h of fermentation with the studied LAB strains. The measured values for all strains of the same LAB species are pooled together. Data are presented as the mean ± SD. Values measured for the unfermented flour are represented by lines.

The data on the dynamic viscosity of the cereal flours fermented for 24 h with the studied LAB strains are presented in Figure 4. In general, all studied samples, both unfermented and fermented, demonstrated typical non-Newtonian behavior characterized by shear thinning—the samples’ viscosity decreased with the increase in shear rate from 12.9 to 64.6 s^−1^. Only in the case of the wheat flour, there was no change (*p* > 0.05) in flour viscosity after fermentation. In the case of the rice and oat flours, the strains of *Lb. paracasei*, *Lb. helveticus*, and *Lc. lactis* significantly (*p* < 0.05) increased flour viscosity by 13–26% after fermentation. At the same time, the fermentation with strains of *Lb. delbrueckii* significantly (*p* < 0.05) decreased the viscosity of the rice and oat flours by ~5% and ~40%, respectively. The strains of *Str. thermophilus* significantly (*p* < 0.05) decreased the viscosity of the rice flour by ~5% and significantly (*p* < 0.05) increased the viscosity of the oat flour by ~21%.

It is generally known that for suspensions of cereal flour, the amylose/amylopectin ratio in a sample and its viscosity are negatively correlated. Amylopectin is a main component of starch granules responsible for their swelling and, consequently, for the viscosity of flour suspensions, and amylose tends to bind to amylopectin and reduce its swelling ability [90,91]. Thus, the changes observed in our experiments in the viscosity of the fermented flour samples may indicate a change in the amylose/amylopectin ratio during the hydrolysis of starch granules by LAB enzymes.

Since both increases and decreases in viscosity were observed during fermentation with different LABs, the change in the amylose/amylopectin ratio seemed to depend on the individual set of amylolytic enzymes of each LAB and their substrate specificity. Similar to our findings, Zhang et al. [86] showed that the viscosity of fermented proso millet flour depends on the used LAB species; in their work, *Lb. plantarum* and *Lb. amylovorus* significantly decreased flour viscosity, while no changes in flour viscosity were observed after fermentation with *Lb. fermentum*. In addition, the decrease in the viscosity of oat flour fermented with *Lb. delbrueckii* may be associated with the hydrolysis of *β*-glucan, the main non-starch polysaccharide of oat flour that largely determines its rheological parameters [92].

### 3.5. Degree of Proteolysis and Antioxidant Capacity

**Figure 5:**
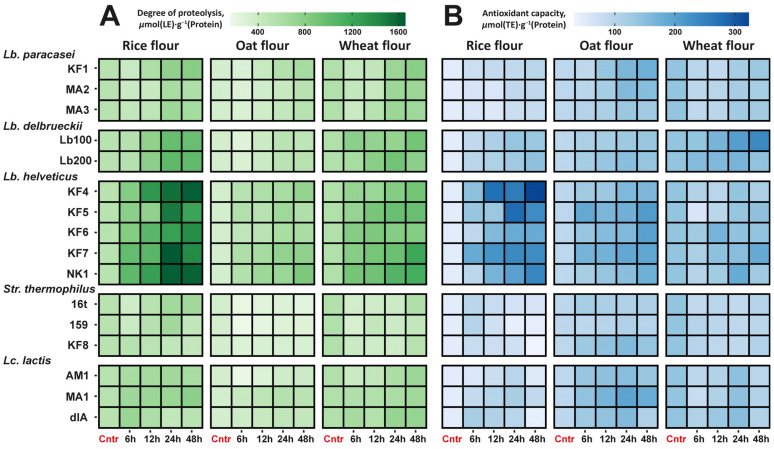
Changes in degree of proteolysis (**A**) and antioxidant capacity (**B**) during the fermentation of cereals with the studied LAB strains.

In the current study, the degree of proteolysis that roughly indicates the amount of peptides in the fermentation medium was constantly increasing during fermentation (Figure 5A). The initial degrees of proteolysis were 511 ± 6, 337 ± 4, and 557 ± 7 µmol(LE)·g^−1^ (Protein) for the rice, oat, and wheat flours, respectively. After 48 h of fermentation, the degree of proteolysis increased by 2–3 times on average. Thus, the total consumption of free proteins from the medium was accompanied by an increase in the amount of low-molecular-weight peptides. This situation is a consequence of the peculiarities of the LAB proteolytic system. During their growth, LABs secrete cell envelope proteinases (CEPs), which carry out the extracellular degradation of proteins into low-molecular-weight peptides. After this, a specific transport system delivers the peptides into the cell for further breakdown into amino acids [70,93].

Although some intra-specific variation in the degree of proteolysis was observed, it was relatively small compared to the inter-specific variation. Irrespective of the type of flour, the highest degree of proteolysis was detected for the flour fermented by the strains of *Lb. helveticus*, while the lowest degree of proteolysis was detected for the flour fermented by the strains of *Str. thermophilus*.

**Figure 6:**
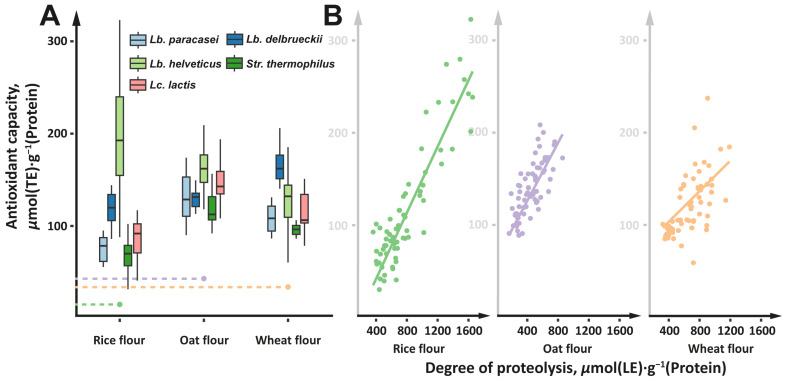
(**A**) Changes in antioxidant capacity induced by the fermentation of cereal flour with the studied LAB strains. The measured values for all strains of the same LAB species are pooled together. Values measured for the unfermented flour are represented by dashed lines with a dot at the end. (**B**) Correlation between the degree of proteolysis and the antioxidant capacity of the flour fermented by the studied LAB strains.

The antioxidant capacity of the fermented flour was measured as the ability to scavenge free radicals from the environment. As with the degree of proteolysis, the antioxidant capacity was constantly increasing during the fermentation process (Figure 5B). The antioxidant capacities of the unfermented rice, oat, and wheat flour were 47 ± 4, 92 ± 6, and 140 ± 11 µmol (TE)·g^−1^ (Protein), respectively. After 48 h of fermentation, the antioxidant capacity increased by an average of 1.5–5 times. In the ability of LABs to increase the antioxidant capacity of the fermented flour, species specificity prevailed over strain specificity (Figure 6A). The highest antioxidant capacity was detected for the flour fermented by the strains of *Lb. helveticus*, while the lowest antioxidant capacity was detected for the flour fermented by the strains of *Str. thermophilus*. Moreover, antioxidant capacity well correlated (R^2^ from 0.9 to 0.6, *p* < 0.05) with the degree of proteolysis (Figure 6B). In contrast, no correlation was observed between antioxidant capacity and the content of free phenolic compounds.

The correlation between the degree of proteolysis and the antioxidant capacity observed for fermented cereal flour has previously been reported for fermented milk [94]. Probably, for both fermentations of milk and cereal flour, these correlations are of the same nature. The antioxidant properties of peptides are relatively sequence-independent; in order to have good antioxidant activity, peptides should be short (up to 20 amino acids) and contain a high proportion of hydrophobic amino acids [26,95,96]. Because of this sequence independence, the higher the degree of proteolysis in a fermented product, the greater the total pool of antioxidant peptides the product will contain, virtually regardless of the nature of the specific proteins being hydrolyzed and the substrate specificity of the proteolytic system of a particular LAB strain.

### 3.6. Angiotensin-Converting Enzyme Inhibitory Activity

The potential antihypertensive activity of the fermented flours was assessed by their ability to inhibit the angiotensin-converting enzyme (ACE) in vitro. As an indispensable part of the renin–angiotensin–aldosterone system (RAAS), the ACE converts angiotensin I into octopeptide angiotensin II, the action of which causes an increase in blood pressure through the narrowing of blood vessels and the stimulation of water reabsorption into the blood [97,98]. Currently, the use of inhibitors of the RAAS is the recommended first-line evidence-based therapy for patients with arterial hypertension [99,100,101].

For all fermented flour, the ACE-inhibitory activity was expressed as the half-maximal inhibitory concentration (IC_50_) measured at 24 h of fermentation (Table 3). As with the antioxidant capacity, a general species-specific trend was observed, and strain-specific outliers were rare. Fermentation with the strains of *Lb. paracasei* and *Lb. delbrueckii* significantly (*p* < 0.05) increased the ACE-inhibitory activity (i.e., decreased the value of IC_50_) of the oat and wheat flours, while the ACE-inhibitory activity of the rice flour did not change. Fermentation with the strains of *Lb. helveticus*, *Str. thermophilus*, and *Lc. lactis* significantly (*p* < 0.05) increased the ACE-inhibitory activity of all types of flour; the only exception was *Lb. helveticus* NK1, which significantly (*p* < 0.05) increased the ACE-inhibitory activity of oat flour only. The greatest changes in ACE-inhibitory activity, six-fold compared to the control, were observed for oat flour, and for rice and wheat flour, the ACE-inhibitory activity increased no more than three-fold.

In contrast to the antioxidant capacity, a correlation between ACE-inhibitory activity and the degree of proteolysis was absent. The highest ACE-inhibitory activity was demonstrated by the rice flour fermented with the strains of *Str. thermophilus*, and these fermented flour samples had the lowest degree of proteolysis compared to the others. This can be explained by the fact that the ACE-inhibitory activity of peptides is highly sequence-specific, since they have to perform competitive inhibition at the catalytic sites of the ACE [96]. Consequently, the specificity of the LAB proteolytic system and the nature of hydrolysable proteins are of paramount importance for the production of ACE-inhibitory peptides, and in the produced peptide pool, only a small fraction of peptides, if any, will exhibit ACE-inhibitory activity independently from the degree of hydrolysis.

It is interesting to compare data on the antioxidant capacity and ACE-inhibitory activity of fermented cereal flour and cow’s milk obtained for the same LAB strains [45,94,102,103]. For all LAB strains, the IC_50_ values of the fermented cereal flours were orders of magnitude lower than those of the fermented cow’s milk (Table 3). This makes fermented cereal-based beverages a promising base for the production of functional foods, the regular consumption of which will alleviate, to some extent, symptoms of primary hypertension.

## 4. Conclusions

The global market for cereal-based dairy alternatives is constantly growing, and the diversity of existing local foods produced via cereal fermentation provides an important opportunity for the development of new types of commercial cereal-based products. Currently, high standards of food production require the transition from spontaneous fermentation to more controlled starter cultures with certain properties and compositions. Although there is an extensive amount of information on both single- and mixed-strain starters for dairy-based products, little is known about the fermentation processes of cereals. In our experiments, it has been demonstrated that the pure LAB cultures commonly used for dairy production are suitable for cereal fermentation, regardless of the specific strain used. The obtained cereal-based fermented products had desirable concentrations of potentially probiotic LABs (10^7^–10^8^ CFU·mL^−1^), and the *Lb. paracasei*–rice flour system was found to be the best for obtaining probiotic-enriched beverages. In terms of organoleptic properties, it is worth noting that the strains of *Lc. lactis* imparted a sweetish taste to the fermented flour, and the strains of *Str. thermophilus* produced a fresh fruity aroma. The most pronounced change in viscosity was observed during the fermentation of oat flour—the strains of *Lc. lactis* significantly increased flour viscosity, and the strains of *Lb. delbrueckii* decreased it. In terms of functional properties, the most pronounced increase in antioxidant capacity was observed for the *Lb. helveticus*–rice flour, *Lb. helveticus*–oat flour, *Lc. lactis*–oat flour, and *Lb. delbrueckii*–wheat flour systems. The most pronounced increase in ACE-inhibitory activity was observed for the fermentations with *Str. thermophilus*, regardless of the flour type. Thus, the data obtained here make it possible to create combined starters for each type of studied flour in order to obtain products with specified organoleptic and functional properties. However, it is worth noting that combined starters require separate study and further optimization.

## Figures and Tables

**Table 1:** The main composition of the flours according to the supplier specifications.

Nutrient	Content, wt%		
	Rice Flour	Oat Flour	Wheat Flour
Protein	5.5	12.0	11.7
Fat	0.8	6.0	1.83
Carbohydrates	81.2	66.0	59.6
Dietary fibers	2.5	4.5	5.3
Starch and dextrins	79.0	63.5	nd
Free sugar (mono- and disaccharides)	0.1	1.0	nd

nd—no data.

**Table 2:** Strains of lactic acid bacteria that were used in this study.

Strain	Isolation Source	BioProject No.
*Lb. paracasei* KF1	Kefir grains	PRJNA824719
*Lb. paracasei* MA2	Mahewu	PRJNA736961
*Lb. paracasei* MA3	Mahewu	PRJNA824719
*Lb. delbrueckii* Lb100	Yogurt	PRJNA736961
*Lb. delbrueckii* Lb200	Sour milk	PRJNA824719
*Lb. helveticus* KF4	Kefir grains	PRJNA824719
*Lb. helveticus* KF5	Kefir grains	PRJNA824719
*Lb. helveticus* KF6	Kefir grains	PRJNA824719
*Lb. helveticus* KF7	Kefir grains	PRJNA824719
*Lb. helveticus* NK1	Infant feces	PRJNA736961
*Str. thermophilus* 16t	Sour milk	PRJNA824719
*Str. thermophilus* 159	Orange leaves	PRJNA736961
*Str. thermophilus* KF8	Kefir grains	PRJNA824719
*Lc. lactis* AM1	Amasi	PRJNA824719
*Lc. lactis* MA1	Mahewu	PRJNA824719
*Lc. lactis* dlA	Sour cream	PRJNA736961

**Table 3:** Angiotensin-converting enzyme-inhibitory activity (expressed as half-maximal inhibitory concentration, IC_50_) of the cereal flour fermented for 24 h by different strains of lactic acid bacteria.

	IC_50_, mg (Protein)·mL^−1^			
	Rice Flour (3%)	Oat Flour (3%)	Wheat Flour (3%)	Milk ^1^
Unfermented control	0.18 ± 0.02	0.61 ± 0.03	0.23 ± 0.02	
**Range from min to max**	**0.16** **–0.21**	**0.57** **–0.65**	**0.19** **–0.26**	**7.1** **–9.0**
**Strains of *Lb. paracasei***				
KF1	0.16 ± 0.01	0.36 ± 0.04	0.28 ± 0.03	
MA2	0.22 ± 0.03	0.40 ± 0.05	0.23 ± 0.01	
MA3	0.25 ± 0.02	0.34 ± 0.02	0.28 ± 0.03	
**Range from min to max**	**0.14** **–0.28**	**0.29** **–0.42**	**0.20** **–0.31**	**2.1** **–2.5**
**Strains of *Lb. delbrueckii***				
Lb100	0.22 ± 0.02	0.40 ± 0.07	0.14 ± 0.02	
Lb200	0.26 ± 0.06	0.33 ± 0.03	0.17 ± 0.01	
**Range from min to max**	**0.19** **–0.35**	**0.28** **–0.50**	**0.10** **–0.19**	**1.0** **–2.2**
**Strains of *Lb. helveticus***				
KF4	0.11 ± 0.01	0.12 ± 0.02	0.08 ± 0.01	
KF5	0.12 ± 0.02	0.23 ± 0.03	0.12 ± 0.02	
KF6	0.12 ± 0.01	0.22 ± 0.01	0.11 ± 0.02	
KF7	0.10 ± 0.03	0.21 ± 0.02	0.10 ± 0.03	
NK1	0.17 ± 0.03	0.17 ± 0.01	0.30 ± 0.05	
**Range from min to max**	**0.06** **–0.22**	**0.15** **–0.28**	**0.06** **–0.36**	**0.6** **–2.0**
**Strains of *Str. thermophilus***				
16t	0.05 ± 0.02	0.14 ± 0.02	0.14 ± 0.02	
159	0.06 ± 0.01	0.15 ± 0.03	0.05 ± 0.01	
KF8	0.05 ± 0.01	0.11 ± 0.01	0.14 ± 0.03	
**Range from min to max**	**0.03** **–0.08**	**0.09** **–0.21**	**0.04** **–0.19**	**1.0** **–1.6**
**Strains of *Lc. lactis***				
AM1	0.19 ± 0.03	0.10 ± 0.02	0.13 ± 0.01	
MA1	0.09 ± 0.02	0.23 ± 0.02	0.14 ± 0.03	
dlA	0.07 ± 0.01	0.20 ± 0.05	0.10 ± 0.04	
**Range from min to max**	**0.05** **–0.23**	**0.07** **–0.27**	**0.06** **–0.20**	**1.7** **–3.5**

^1^ Data were taken from the previous publications on the strains used in this study [45,94,102,103].

## Data Availability

The original contributions presented in the study are included in the article, further inquiries can be directed to the corresponding author.
